# Supplementary material for: 1H-NMR Metabolomics as a Tool for Winemaking Monitoring
Source: Molecules. 2021 Nov 9;26(22):6771. doi: 10.3390/molecules26226771 (PMC8621607; doi:10.3390/molecules26226771)
Supplement: Supplementary file 1 [file molecules-26-06771-s001.zip › molecules-1440327-supplementary.pdf]

Supplementary materials

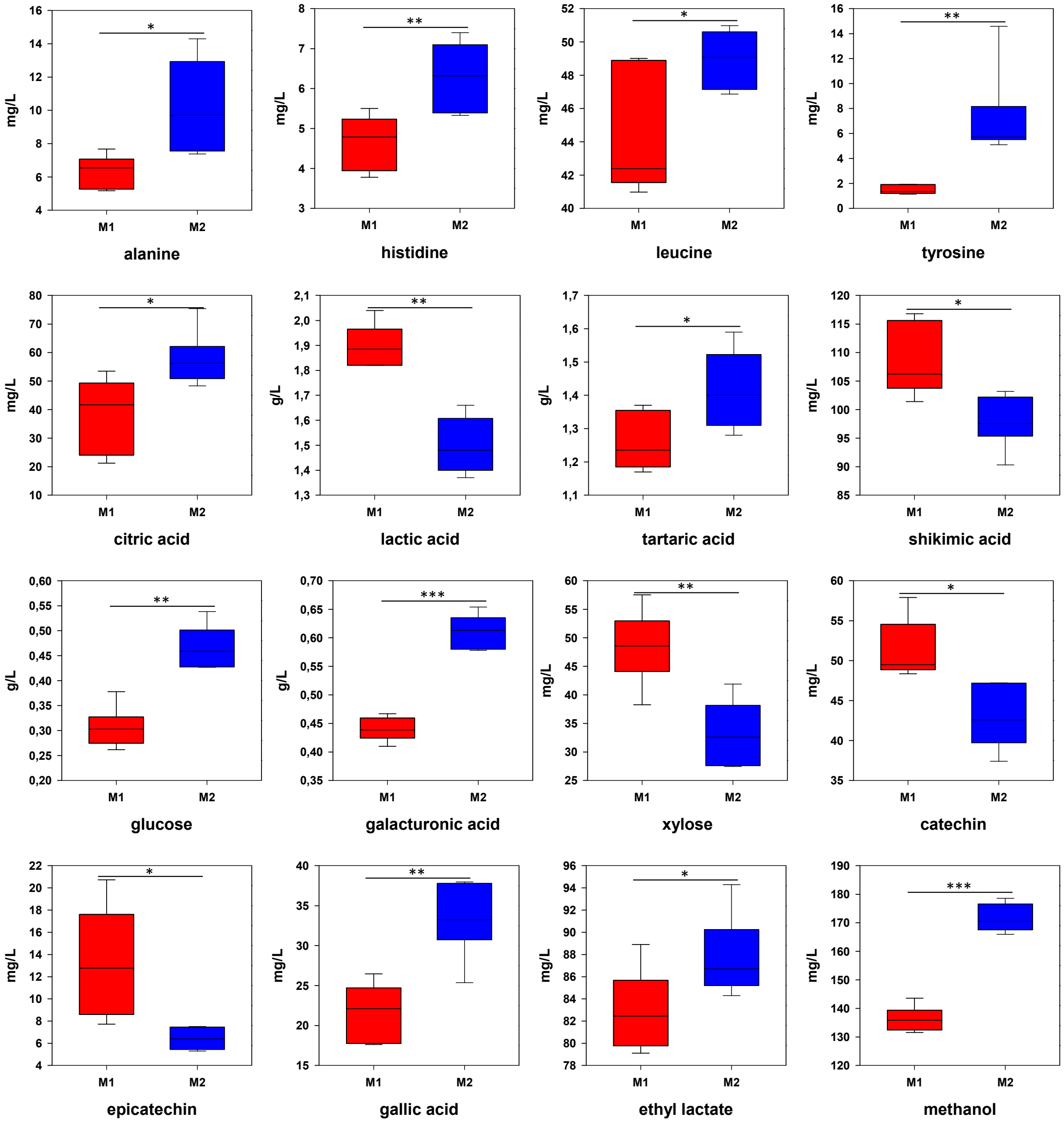

Figure S1. Boxplots of 16 most discriminant wine constituents based on ^{1}H-NMR analysis of wines produced from grapes harvested under-maturity (M1) and at maturity (M2). The significance in the difference was calculated by ANOVA followed by Tukey's multiple comparison test (indicated as *p < 0.05, **p < 0.01, ***p < 0.001).

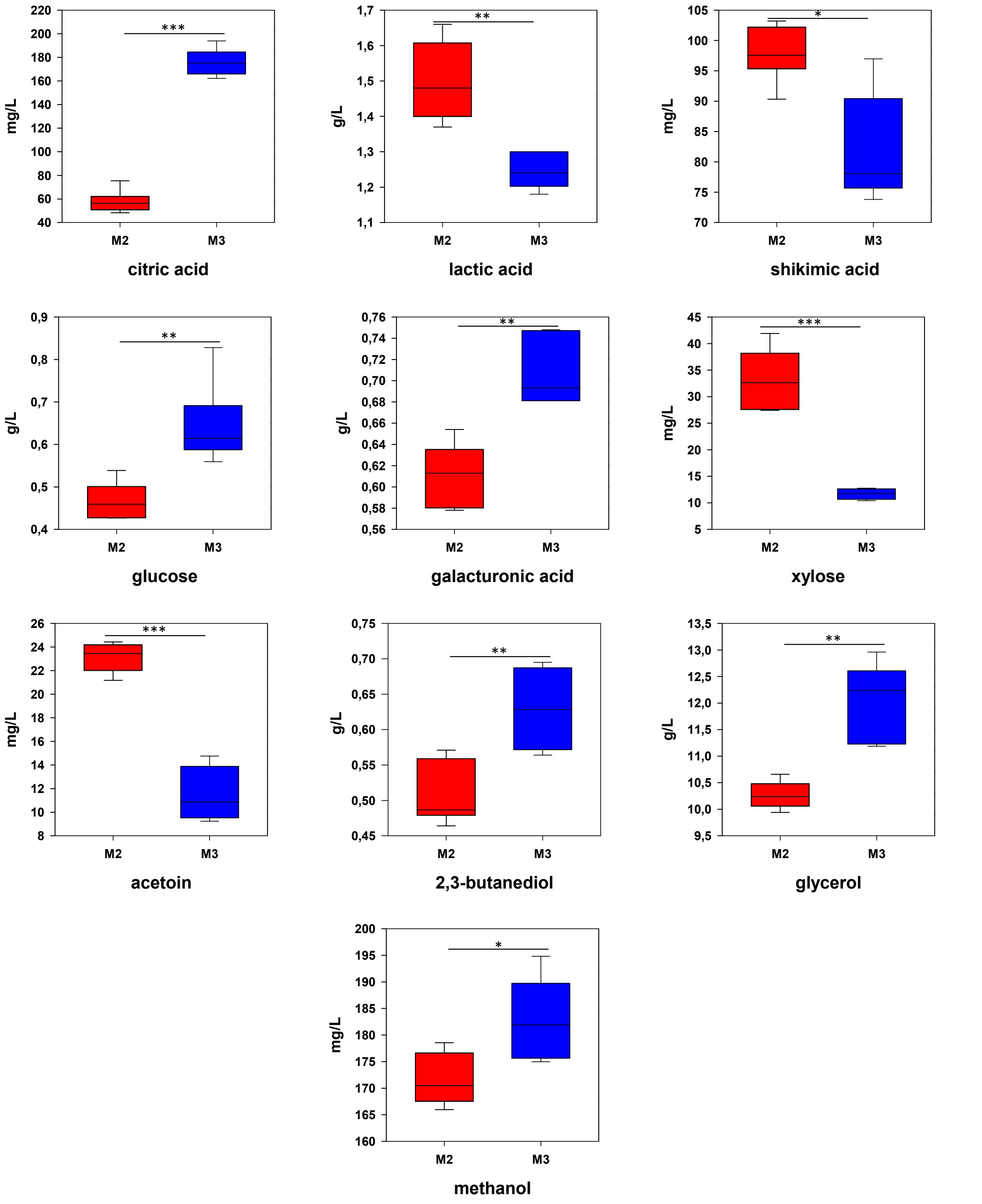

Figure S2. Boxplots of 10 most discriminant wine constituents based on ^{1}H-NMR analysis of wines produced from grapes harvested at maturity (M2) and at over-maturity (M3). The significance in the difference was calculated by ANOVA followed by Tukey's multiple comparison test (indicated as *$p < 0.05$, **$p < 0.01$, ***$p < 0.001$).

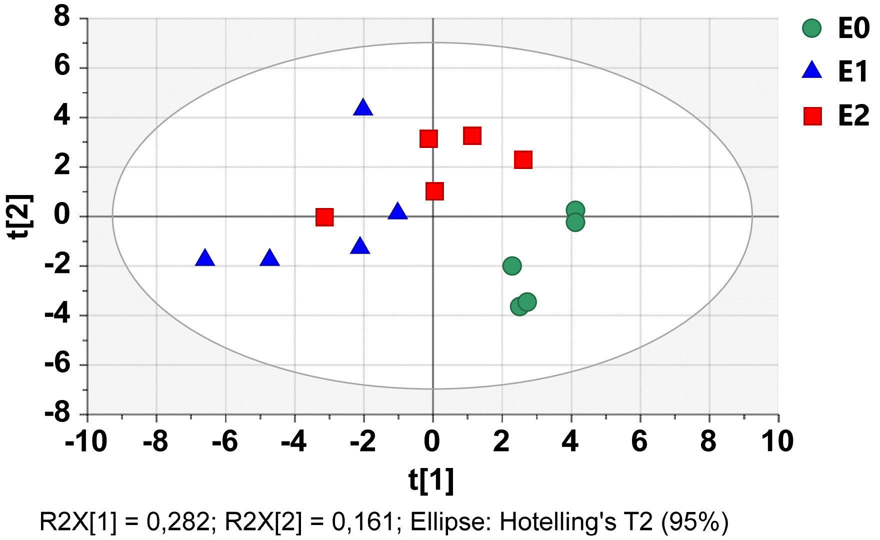

A

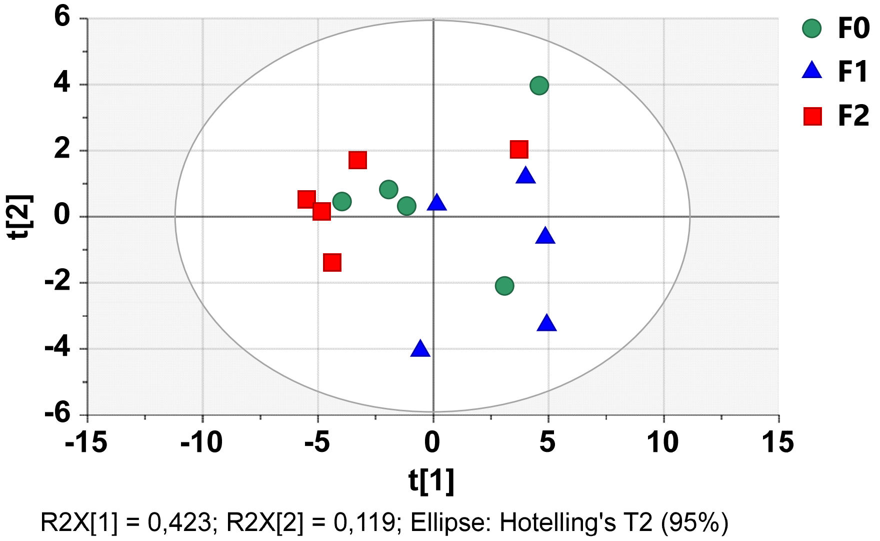

B

Figure S3. PCA score plots from ^{1}H-NMR spectra of wine samples. (A) Wine samples treated by different enzymes (E0: untreated; E1: enzyme 1; E2: enzyme 2); (B) wine samples treated by different finings (F0: untreated; F1: fining 1; F2: fining 2).

Table S1. Grape pH, sugar content, total acidity and malic acid contents at the three different harvest times (M1: under-maturity; M2: maturity; M3: over-maturity).

**Physiological parameters**	**M 1**	**M 2**	**M 3**
pH	3.49	3.51	3.46
sugar content (g/L)	192.9	190.5	212.5
Total acidity (g/L)	3.06	3.53	3.51
malic acid (g/L)	2.25	1.88	1.59

Table S2. Misclassification table of OPLS analysis of wine samples from grapes at different stages of maturity (M1: under-maturity; M2: maturity; M3: over-maturity).

	**Members**	**Correct**	**M1**	**M2**	**M3**	**No class**
**M1**	6	100%	6	0	0	0
**M2**	6	100%	0	6	0	0
**M3**	6	100%	0	0	6	0
**No class**	0		0	0	0	0
**Total**	18	100%	6	6	6	0
**Fisher's prob.**	3.5×10^{-7}					

Table S3. OPLS-DA model parameters for discrimination between M1 (under-maturity), M2 (maturity) and M3 (over-maturity). OPLS-DA models were controlled using cross-validation for discrimination between the three groups, and 100 permutation tests of corresponding validation plots.

	**R^2X(cum)**	**R^2Y(cum)**	**Q^2(cum)**	**R^2 intercepts**	**Q^2 intercepts**
**M2 vs M3**	0.448	0.994	0.951	0.763	-0.453
**M1 vs M2**	0.615	0.975	0.873	0.991	-0.38

Table S4. Misclassification table of OPLS analysis of wine samples treated by enzymes (E0: control; E1: enzyme 1; E2: enzyme 2).

	Members	Correct	E0	E1	E2	No class
**M1**	5	100%	5	0	0	0
**M2**	5	100%	0	5	0	0
**M3**	5	100%	0	0	5	0
**No class**	0		0	0	0	0
**Total**	15	100%	5	5	5	0
**Fisher's prob.**	7.9×10^{-6}					

Table S5. Misclassification table of OPLS analysis of wine samples treated by fining agents (F0: control; F1: fining 1; F2: fining 2).

	Members	Correct	F0	F1	F2	No class
**M1**	5	100%	5	0	0	0
**M2**	5	100%	0	5	0	0
**M3**	5	100%	0	0	5	0
**No class**	0		0	0	0	0
**Total**	15	100%	5	5	5	0
**Fisher's prob.**	7.9×10^{-6}					
